# Differential Regulation of miRNA and Protein Profiles in Human Plasma-Derived Extracellular Vesicles via Continuous Aerobic and High-Intensity Interval Training

**DOI:** 10.3390/ijms26031383

**Published:** 2025-02-06

**Authors:** Zhenghao Wang, Yiran Ou, Xinyue Zhu, Ye Zhou, Xiaowei Zheng, Meixia Zhang, Sheyu Li, Shao-Nian Yang, Lisa Juntti-Berggren, Per-Olof Berggren, Xiaofeng Zheng

**Affiliations:** 1 Department of Endocrinology and Metabolism, Research Center for Islet Transplantation, West China Hospital, Sichuan University, Chengdu 610041, China; zhenghao.wang@ki.se (Z.W.); yrou2001@163.com (Y.O.); xinyuezhu68@wchscu.cn (X.Z.); zhouye202501@163.com (Y.Z.); xiaowei.zheng@ki.se (X.Z.); lisheyu@gmail.com (S.L.); per-olof.berggren@ki.se (P.-O.B.); 2 The Rolf Luft Research Center for Diabetes and Endocrinology, Karolinska Institutet, SE-17176 Stockholm, Sweden; shao-nian.yang@ki.se (S.-N.Y.); lisa.juntti-berggren@ki.se (L.J.-B.); 3 Department of Molecular Medicine and Surgery, Karolinska Institutet, SE-17177 Stockholm, Sweden; 4 Research Laboratory of Macular Disease, Department of Ophthalmology, West China Hospital, Sichuan University, Chengdu 610041, China; zhangmeixia@scu.edu.cn

**Keywords:** exercise, extracellular vesicles, multi-omic approaches, organ crosstalk

## Abstract

Both continuous aerobic training (CAT) and high-intensity interval training (HIIT) are recommended to promote health and prevent diseases. Exercise-induced circulating extracellular vesicles (EX-EVs) have been suggested to play essential roles in mediating organ crosstalk, but corresponding molecular mechanisms remain unclear. To assess and compare the systemic effects of CAT and HIIT, five healthy male volunteers were assigned to HIIT and CAT, with a 7-day interval between sessions. Plasma EVs were collected at rest or immediately after each training section, prior to proteomics and miRNA profile analysis. We found that the differentially expressed (DE) miRNAs in EX-EVs were largely involved in the regulation of transcriptional factors, while most of the DE proteins in EX-EVs were identified as non-secreted proteins. Both CAT and HIIT play common roles in neuronal signal transduction, autophagy, and cell fate regulation. Specifically, CAT showed distinct roles in cognitive function and substrate metabolism, while HIIT was more associated with organ growth, cardiac muscle function, and insulin signaling pathways. Interestingly, the miR-379 cluster within EX-EVs was specifically regulated by HIIT, involving several biological functions, including neuroactive ligand–receptor interaction. Furthermore, EX-EVs likely originate from various tissues, including metabolic tissues, the immune system, and the nervous system. Our study provides molecular insights into the effects of CAT and HIIT, shedding light on the roles of EX-EVs in mediating organ crosstalk and health promotion.

## 1. Introduction

Exercise has long been recommended as a fundamental strategy to improve physical and mental fitness, which play pivotal roles in the prevention and treatment of various diseases, including cardiometabolic diseases, neurological diseases, sarcopenia, and cancer [1]. Exercise can be primarily categorized into aerobic and anaerobic exercises [2]. Aerobic exercise, which includes continuous aerobic training (CAT), involves continuous moderate-intensity activity and typically improves cardiorespiratory fitness. In contrast, anaerobic exercise, which includes sprinting, high-intensity interval training (HIIT), and power-lifting, involves short bursts of high-intensity activity and primarily focuses on promoting strength, power, and speed [3]. Despite their distinct features, both aerobic and anaerobic exercises present similar beneficial roles and result in comparable enhancements in glycemic control and mitochondrial function [4]. In general, exercise is believed to trigger a major challenge to cellular, tissue, and whole-body homeostasis, where a myriad of epigenetic, metabolic, and transcriptional regulations are involved in the adaptive responses to exercise [5]. During this metabolic activity, the intensity, frequency, and duration of exercise determine the overall metabolic and molecular responses. In particular, aerobic and anaerobic exercise represent distinct ends of the exercise continuum, resulting in substrate-level oxidative phosphorylation and phosphorylation, respectively [6]. Interestingly, even a single bout of exercise elicits acute adaptive responses, while regular periods of exercise promote long-term adaptation processes [2]. It has been suggested that exercise-mediated beneficial effects are at least partially attributed to tissue crosstalk [7]. In fact, exercise stimulates numerous cells and tissues, including immune cells, skeletal muscle, liver, adipose tissue, brain, and bone, to secret bioactive molecules into the circulation, which, in turn, act in an autocrine, paracrine, or endocrine manner to promote positive outcomes [8]. However, the underlying molecular mechanisms responsible for exercise-mediated tissue crosstalk and its potential effects remain largely unexplored.

Extracellular vesicles (EVs), a diverse group of lipid bilayer vesicles that are secreted by almost all cells, contain bioactive molecules, including proteins, nucleic acids, and lipids, and play essential roles in intercellular and interorgan communication [9,10]. Numerous studies have reported that a single bout of exercise can rapidly trigger a significant increase in the amount of circulating EVs in both humans and rodents [11], indicating that exercise may stimulate the release of EVs from various tissues into circulation. Furthermore, it has been shown that the circulating EV content can be altered by exercise [12], highlighting the critical roles of EVs in mediating tissue crosstalk during exercise. For instance, a significant number of circulating EV proteins were found to be regulated by exercise and extensively participate in different biological processes, such as glycolysis and immune regulation [8,13]. MiRNA, a class of non-coding RNAs with a length of about 20–25 nucleotides, serves as one of the most important active components of EVs. MiRNAs are involved in post-transcriptional regulation of more than 60% of protein-coding genes in mammals, which play essential roles in various physiological and pathological processes [14]. Interestingly, exercise-regulated circulating EV miRNAs have been demonstrated to mediate health-promoting processes such as cardiovascular protection and white adipose tissue browning [14,15]. Although great efforts have been made to elucidate the roles of EX-EVs, current studies have focused only on a single exercise mode with a single type of omics analysis, thereby limiting our current understanding. A comprehensive comparison of different types of exercise modalities with multi-omics integration analysis of EX-EVs is needed.

In this study, we analyzed and compared the systemic effects of a single bout of CAT and HIIT by performing an integrated analysis of differentially regulated proteins and miRNAs within circulating EVs. Our aim was to elucidate the molecular mechanisms underlying the roles of EVs in mediating organ crosstalk and health promotion under different exercise modes.

## 2. Results

### 2.1. General Characterizations of Exercise Participants and Their Plasma EVs

**Figure 1:**
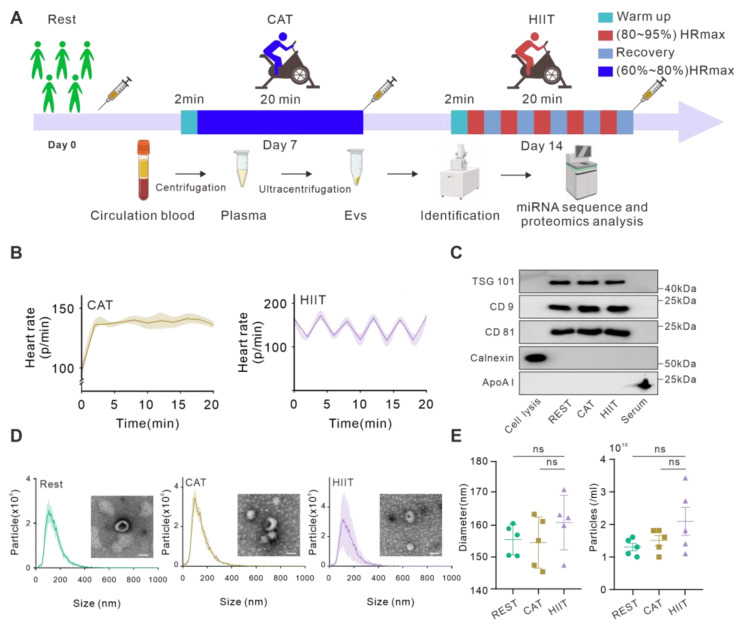
Obtention and characterization of human plasma-derived EVs before and after two types of exercise. (**A**) Schematic illustration of the study design. In brief, five healthy volunteers who underwent acute CAT and HIIT at an interval of 7 days were enrolled in the experiment. Blood samples were collected in heparin-coated blood collection tubes at rest or immediately after each training session, followed by centrifugation at 1600× *g* for 10 min at 4 °C to separate plasma. The plasma fraction was then subjected to ultracentrifugation for EV isolation, and aliquots from the same EV preparations were subsequently used for EV characterization, proteomics, and miRNA profile analysis, respectively. (**B**) Real-time heart rate monitoring of volunteers during CAT and HIIT (n = 5). (**C**) Western blot analysis of EV markers. (**D**) Evaluation of morphology and size distribution of EVs via TEM and NTA, respectively. Scale bar = 50 nm. (**E**) Quantification of the average diameter and concentration of plasma EVs obtained from the indicated groups (n = 5); ns = non-significant difference.

Five healthy individuals participated in the study, and their clinical characteristics are provided in Table S1. All the participants underwent CAT and HIIT at an interval of 7 days. Blood samples were collected at rest or immediately after each training session, and plasma EVs were extracted and subjected to proteomics and miRNA profile analysis (Figure 1A). As shown in Figure 1B, the target heart rate was maintained at around 60–80% of HRmax during CAT, while HIIT elicited 85% of HRmax interspersed with 2 min of active recovery at 70% of HRmax. To determine the characteristics of the isolated plasma EVs, we evaluated the presence of the commonly used EV markers using WB, morphology using TEM, and size distribution using NTA, as recommended [16]. Typical EV markers, such as TSG101, CD9, and CD81, could be detected, while the negative EV marker Calnexin or plasma marker apolipoprotein AI was undetectable in the EV samples (Figure 1C), indicating that the obtained plasma EVs were free of blood cells and plasma. TEM revealed the typical saucer-like morphology of EVs obtained from all groups (Figure 1D). Furthermore, plasma EVs isolated from each group displayed comparable size distributions with mean sizes of 156.1 ± 2.1 nm for the REST group, 155.2 ± 3.6 nm for the CAT group, and 161.5 ± 3.8 nm for the HIIT group, respectively (Figure 1D,E). An interesting trend toward an increase in the concentration of plasma EVs was observed in the HIIT group compared to the REST group, although the difference was not statistically significant (Figure 1E).

### 2.2. Effects of CAT and HIIT on miRNA Profiles in Human Plasma-Derived EVs

**Figure 2:**
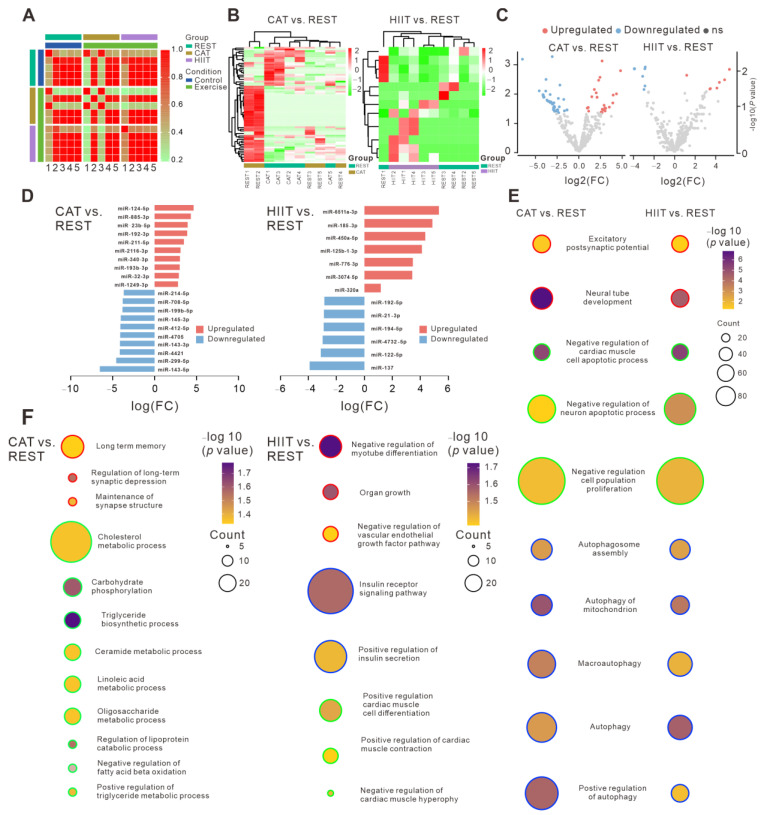
miRNA profiles and functional enrichment analysis of plasma EVs. (**A**) Correlation matrix analysis of miRNA signatures of the indicated groups. (**B**) Two-way hierarchical clustering heatmaps of DE EV miRNAs in the CAT vs. REST comparison (left panel) and in the HIIT vs. REST comparison (right panel). (**C**) Volcano plots showing DE EV miRNAs in the CAT vs. REST comparison (left panel) and in the HIIT vs. REST comparison (right panel). (**D**) Bar plots illustrating the most upregulated and downregulated miRNAs in the CAT vs. REST comparison (left panel) and in the HIIT vs. REST comparison (right panel). (**E**) Commonly enriched GO biological processes of target genes of the DE miRNAs in both CAT vs. REST comparison and the HIIT vs. REST comparison. Same colors of the bubble contour represent similar biological functions that different GO biological processes involved. (**F**) Differentially enriched GO biological processes of target genes of the DE miRNAs in the CAT vs. REST comparison (left panel) and the HIIT vs. REST comparison (right panel). In each subgroup (CAT vs. REST or HIIT vs. REST), same colors of the bubble contour represent similar biological functions that different GO biological processes involved.

We analyzed and compared miRNA profiles of plasma EVs obtained from REST, CAT, and HIIT groups (Figure S1 and Figure 2). To determine the correlations among different samples, principal component analysis (PCA) (Figure S3) and correlation matrix analysis were performed. Specifically, different miRNA expression profiles were observed not only between the exercise and control groups but also between the CAT and HIIT groups (Figure 2A). A total of 67 DE miRNAs (22 upregulated and 45 downregulated, SI-DE miRNAs) were identified in the CAT group compared to those in the REST group, while 13 DE miRNAs (7 upregulated and 6 downregulated) were identified in the HIIT group compared to those in the REST group (Figure 2B,C). The top 10 most upregulated and downregulated miRNAs in each pairwise comparison are listed in Figure 2D.

Next, the potential target genes of the identified DE miRNAs were predicted. A total of 874,698 and 390,841 target genes were predicted based on RNAhybrid and miRanda, respectively, among which 94,674 target genes overlapped, as shown in the Venn diagram (Figure S4). To elucidate the possible molecular mechanisms connecting the EV miRNA content to the health benefits of two different types of exercise, GO and KEGG pathway enrichment analyses were conducted. Target genes of the DE miRNAs in both CAT and HIIT groups (vs. REST group) were mainly enriched in “nuclear chromatin” for the GO-cellular component (CC) terms (Figure S5A,B) and “DNA-binding transcription factor activity” for GO-molecular function (MF) terms (Figure S5C,D), suggesting that the DE miRNAs in both CAT and HIIT groups are largely involved in the regulation of transcription factors. Among these transcription factors, NEUROG1 [17], SOX12, and SOX13 [18] are shown to be important for neuronal development, RUNX3 [19] is suggested to play key roles in the immune system; TEAD3 and TEAD4 [20] are involved in cell proliferation and differentiation; and KLF11, KLF15 [21], and PPARD [22] are proven to be responsible for the regulation of metabolism. GO analysis further revealed that target genes of the DE miRNAs in the CAT and HIIT groups (vs. the REST group) were commonly enriched in biological processes such as neuronal signal transduction, autophagy, and cell fate regulation (especially for the neuron and cardiomyocytes) to a similar extent (Figure 2E). Furthermore, target genes of the DE miRNAs in the CAT vs. REST group were more specifically enriched in cognitive function and substrate metabolism, while target genes of the DE miRNAs in the HIIT vs. REST group were more specifically enriched in organ growth, cardiac muscle function, and the insulin signaling pathway (Figure 2F). Additionally, KEGG enrichment analysis demonstrated that the most significantly enriched pathways in both CAT and HIIT groups were commonly associated with autophagy and neuronal signal transduction, while the most significantly enriched pathways in CAT and HIIT groups were also specifically associated with substrate metabolism and signal transduction in cardiomyocytes, respectively (Figure S6), which is consistent with the data of GO analysis.

### 2.3. Identification of the Possible Tissue Origin of DE Plasma EV miRNAs

**Figure 3:**
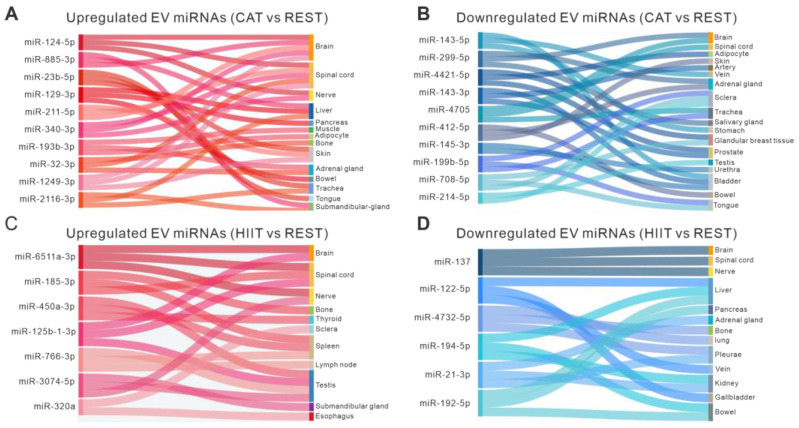
Tissue-specific enrichment analysis of DE EV miRNAs. (**A**,**B**) Sankey diagrams visualizing the tissue origin of the upregulated (**A**) and downregulated (**B**) EV miRNAs in the CAT group compared to REST group. (**C**,**D**) Sankey diagrams visualizing the tissue origin of the upregulated (**C**) and downregulated (**D**) EV miRNAs in the HIIT group compared to REST group.

To assess the contributions of various tissues to the profile of circulating EV miRNAs in response to different types of exercise, tissue-specific enrichment analysis was performed on DE plasma EV miRNAs using the Tissue Atlas. Sankey network diagrams were used to visualize the tissue origin of the most significantly altered EV miRNAs. We found that multiple tissues contributed to the altered expression of EV miRNAs in response to CAT or HIIT. Remarkably, the DE EV miRNAs in both CAT and HIIT groups (vs. the REST group) were found to be enriched in the nervous system (Figure 3A–D), highlighting the involvement of the nervous system during exercise. Interestingly, the upregulated EV miRNAs in the CAT group (vs. the REST group) were suggested to be associated with multiple metabolic tissues, including liver, pancreas, muscle, and adipocytes, while the upregulated EV miRNAs in the HIIT group (vs. the REST group) were shown to be associated with the immune system, such as the spleen and lymph nodes (Figure 3A,C).

### 2.4. Identification of the miR-379 Cluster and miR-154 Family Among HIIT-Regulated Plasma EV miRNAs

**Figure 4:**
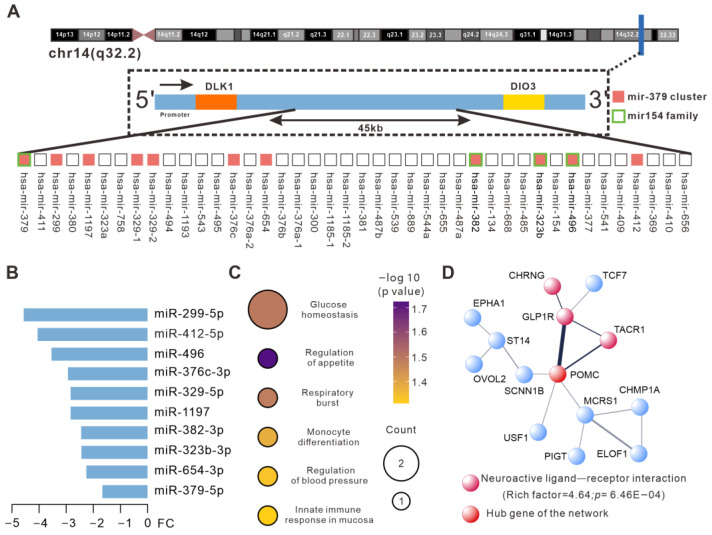
Regulation of the miR-379 cluster and miR-154 family within plasma EVs by HIIT. (**A**) Genomic locations of the miR-379 cluster and miR-154 family. The promoter region is located approximately 19 kb upstream of Chr14MC, which contains the miR-379 cluster and the mir-154 family. The orange squares denote HIIT-regulated miR-379 cluster members, while the green frames denote HIIT-regulated miR-154 family members. (**B**) The bar graph showing fold changes in the expression of HIIT-regulated miR-379 cluster members. (**C**) Enriched GO biological processes of target genes of the DE miRNAs in the HIIT vs. REST comparison. (**D**) PPI analysis of target genes of the DE miRNAs in the HIIT vs. REST comparison. Line thickness indicates the strength of the PPI.

To investigate whether any miRNA cluster in plasma EVs could be regulated by CAT or HIIT, we analyzed the DE EV miRNAs identified in the CAT and HIIT groups (vs. the REST group) using the TAM 2.0 database. Interestingly, the miR-379 cluster, positioned on the chr14q (q32.2) genomic locus, was demonstrated to be specifically regulated by HIIT (FDR < 0.05). Eleven miR-379 cluster members, including miR-299, miR-412, miR-496, miR-376c, miR-329-1, miR-329-2, miR-1197, miR-382, miR-323b, miR-654, and miR-379, were significantly downregulated by HIIT, among which miR-379, miR-382, miR-323b, and miR-496 were also members of the miR-154 miRNA family (Figure 4A,B). To determine the biological roles of the HIIT-regulated miR-379 cluster, the potential target genes of the DE miR-379 cluster members were analyzed via GO enrichment analysis, which revealed that biological processes, including glucose homeostasis, innate immune response in the mucosa, monocyte differentiation, respiratory burst, the regulation of blood pressure, and appetite, were involved (Figure 4C). Furthermore, the STRING database was used to predict the gene interactions among the target genes of the DE miR-379 cluster members. A protein–protein interaction (PPI) network of 14 proteins was identified to be involved, where CHRNG, GLP1R, TACR1, and POMC genes were enriched in the biological process of “neuroactive ligand-receptor interaction” (RF = 4.64 with *p* = 6.46 × 10^−4^), with POMC identified as the hub gene of the network (Figure 4D).

### 2.5. Effects of CAT and HIIT on Proteomic Profiles of Human Plasma-Derived EVs

**Figure 5:**
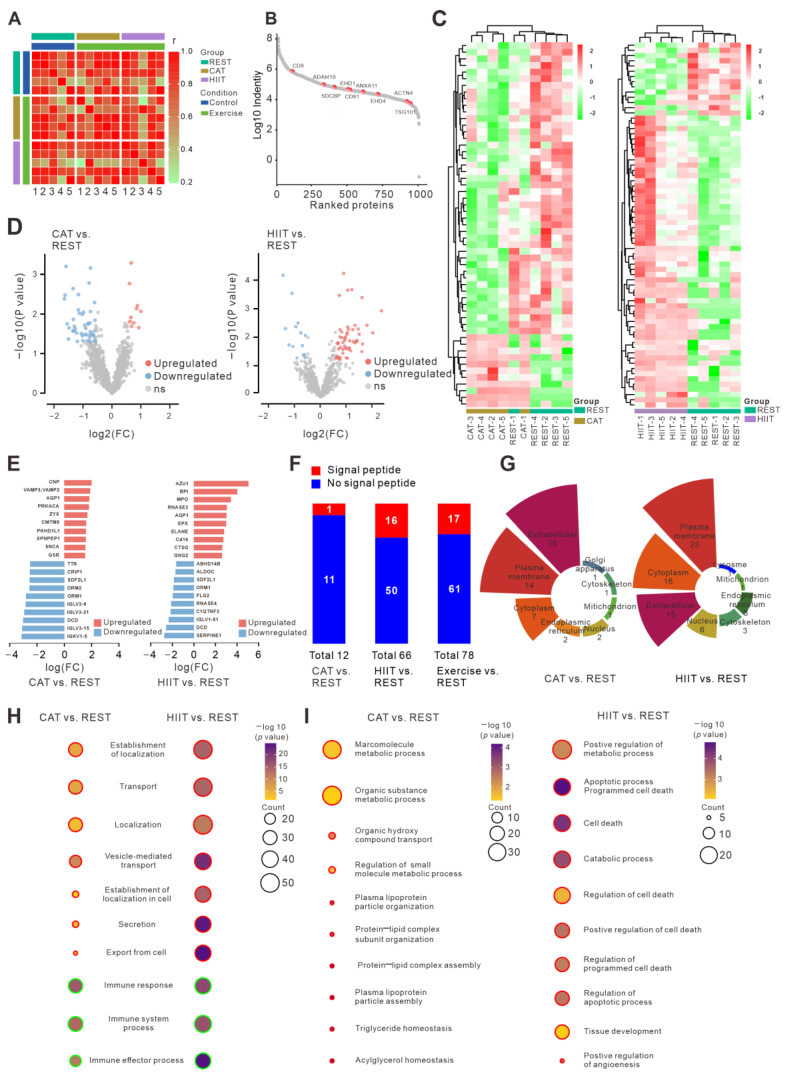
Proteomic profiles and functional enrichment analysis of plasma EVs. (**A**) Correlation matrix analysis of protein signatures of the indicated groups. (**B**) Rank plot of total proteins based on their average log10 intensity. Some of the known EV marker proteins are highlighted in red. (**C**) Two-way hierarchical clustering heatmaps of DE EV proteins in the CAT vs. REST comparison (left panel) and in the HIIT vs. REST comparison (right panel). (**D**) Volcano plots of DE EV proteins in the CAT vs. REST comparison (left panel) and in the HIIT vs. REST comparison (right panel). (**E**) Bar plots of the 10 most upregulated and downregulated proteins in the CAT vs. REST comparison (left panel) and in the HIIT vs. REST comparison (right panel). (**F**) Percentage stacked bar charts showing the presence or absence of a signal peptide sequence of DE EV proteins in the indicated comparisons. (**G**) Prediction of the subcellular localization of DE EV proteins in the CAT vs. REST comparison (left panel) and in the HIIT vs. REST comparison (right panel) using Hum-mPLoc3. (**H**) Commonly enriched GO biological processes of DE EV proteins in the CAT vs. REST comparison and HIIT vs. REST comparison. Same colors of the bubble contour represent similar biological functions that different GO biological processes involved. (**I**) Differentially enriched GO biological processes of DE EV proteins in the CAT vs. REST comparison and the HIIT vs. REST comparison. In each subgroup (CAT vs. REST or HIIT vs. REST), same colors of the bubble contour represent similar biological functions that different GO biological processes involved.

To better understand the biological roles of plasma EVs during two types of exercise, we also analyzed and compared the proteomic profiles of plasma EVs obtained from the REST, CAT, and HIIT groups. A total of 990 EV proteins were quantified in our studies. PCA and correlation matrix analysis revealed a distinct segregation among the three study groups (Figure 5A, Figure S7 and Figure S8). As expected, some of the EV marker proteins were identified (Figure 5B). A total of 55 DE proteins (11 upregulated and 44 downregulated SI-DE proteins) were identified in the CAT group compared to those in the REST group, while 70 DE proteins (56 upregulated and 14 downregulated) were identified in the HIIT group compared to those in the REST group (Figure 5C,D). The top 10 most upregulated and downregulated proteins in each pairwise comparison are listed in Figure 5E.

The signal peptide is a short amino acid sequence located at the N-terminus of a protein, with a length of approximately 13 to 36 amino acid residues. It functions to direct the localization of the protein and is typically cleaved after the protein is transported to its site of function or structural role within a membrane region [23]. Notably, the majority of the DE EV proteins (17 out of 78 in total, with 11 out of 12 in the CAT group and 50 out of 66 in the HIIT group) were shown to not carry signal peptides (Figure 5F). GO pathway enrichment analysis was subsequently conducted to elucidate the potential biological roles of the DE EV proteins. In contrast to the DE EV miRNAs, the DE EV proteins in CAT and HIIT groups (vs. REST group) were commonly involved in vesicle secretion, transport, localization, and immune processes (Figure 5H). Furthermore, CAT-regulated EV proteins were more specifically enriched in the biological process of substrate metabolism, while HIIT-regulated EV proteins were more specifically enriched in the biological process of cell death and survival (Figure 5I), which is in line with the results generated from the DE EV miRNAs. Hum-mPLoc3 was used to predict the subcellular localization of the DE EV proteins, and the results showed that most of these DE EV proteins were originally enriched in the extracellular region, plasma membrane, and cytoplasm (Figure 5G), which was further supported by the results of GO-CC and GO-MF analysis (Figure S9). Moreover, KEGG analysis indicated that the CAT-regulated EV proteins were predominantly associated with hormone synthesis and metabolic pathways, while the HIIT-regulated EV proteins were more strongly associated with immune-related pathways (Figure S10).

### 2.6. Identification of the Possible Tissue Origin of DE Plasma EV Proteins 

**Figure 6:**
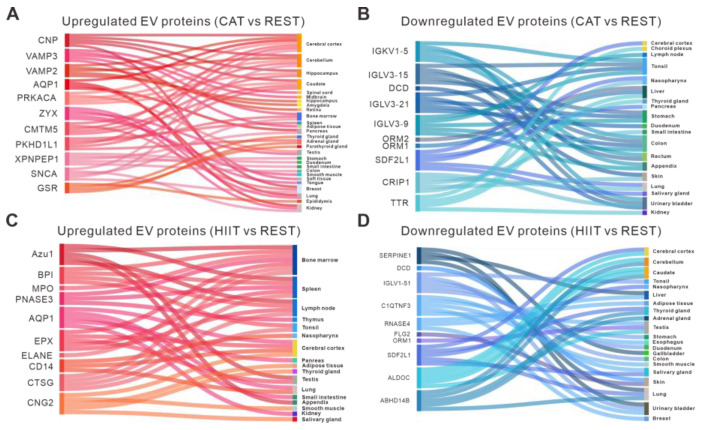
Tissue-specific enrichment analysis of DE EV proteins. (**A**,**B**) Sankey diagrams visualizing the tissue origin of the upregulated (**A**) and downregulated (**B**) EV proteins in the CAT group compared to REST group. (**C**,**D**) Sankey diagrams visualizing the tissue origin of the upregulated (**C**) and downregulated (**D**) EV proteins in the HIIT group compared to REST group.

The Human Protein Atlas was utilized to evaluate the contributions of various tissues to the profile of circulating EV proteins in response to different types of exercise. Similar to the tissue origin of the DE EV miRNAs, numerous tissues were found to contribute to the altered expression of EV proteins in response to CAT or HIIT. Specifically, the DE EV proteins in both CAT and HIIT groups (vs. the REST group) were found to be enriched in the nervous system (Figure 6), which is consistent with the results generated from the DE EV miRNAs (Figure 3). Furthermore, the CAT-upregulated EV proteins were largely enriched in different brain regions, including the cerebral cortex, midbrain, cerebellum, caudate, hippocampus, and amygdala (Figure 6A), while the HIIT-upregulated EV proteins were largely enriched in the immune system, including the bone marrow, spleen, lymph nodes, thymus, tonsils, appendix, and small intestine (Figure 6C).

### 2.7. Integrated Analysis of the DE EV miRNAs and the DE EV Proteins

**Figure 7:**
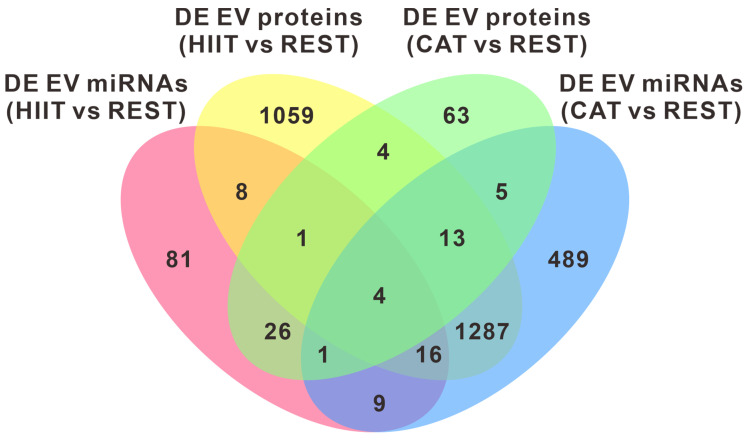
Multivariate Venn diagram of GO terms based on the DE EV proteins and target genes of the DE EV miRNAs in both CAT and HIIT groups.

The roles of EV miRNAs and EV proteins are relatively independent once EVs are released into extracellular space. Instead, EV miRNAs could interplay with EV proteins by regulating their targets in the recipient cells. Therefore, a multivariate Venn diagram was used to overlap the GO terms of the DE EV proteins and target genes of the DE EV miRNAs in both CAT and HIIT groups. As shown in Figure 7 and Table S3, four pathways were found to be co-regulated by EV miRNAs and EV proteins in both CAT and HIIT groups, which were primarily involved in autophagy, cell proliferation, and differentiation. In the CAT group, 23 pathways were found to be co-regulated by EV miRNAs and EV proteins, which were mainly involved in metabolism (lipid and sterol) and the maintenance of cellular homeostasis. Furthermore, in the HIIT group, 29 pathways were found to be co-regulated by EV miRNAs and EV proteins, which were primarily associated with phospholipid metabolism, insulin secretion, and cellular physiological functions. These data further confirmed the overlapping and distinct biological roles of CAT and HIIT.

## 3. Discussion

Exercise triggers the rapid release of EVs into the circulation in both humans and rodents [11,24,25]. In our study, there was an increasing trend in EV concentrations after exercise. Previous studies, especially human studies, have reported inconsistent results regarding whether the amount of EVs increases after exercise. The discrepancy observed in the total particle number may be partly explained by the presence of circulating plasma lipoproteins that cannot be distinguished from EVs by NTA [26]. Nevertheless, the size of EVs remains unmodified regardless of the mode of exercise, isolation method, or measurement technique.

The contents (miRNAs and proteins) of EVs varied from different exercise types in our study. As the concept of “responders” and “non-responders” has been previously proposed in exercise physiology, the degree of responsiveness in individual organs varies depending on the intensity of exercise [27]. Given the regulatory roles of blood flow and organ activation in EV release, the level of exercise intensity may play a pivotal role in determining not only the amount but also the content of circulating EVs [11]. In fact, the differential impacts of low-, moderate-, and high-intensity exercise on the quantity and content of circulating EVs have been demonstrated in rodents [24]. Therefore, examination of plasma-EVs, which are integral constituents of liquid biopsies, may help elucidate genetic and epigenetic biomarkers in the field of exercise physiology.

The physiological impacts of exercise may be partially achieved by circulating EVs through their contained bioactive molecules. During exercise, organ crosstalk can be facilitated by the release of EVs, which are then transported into the circulatory system and delivered to other tissues [28]. Both CAT and HIIT were shown to be involved in neuronal signal transduction, autophagy, and cell death and survival, which is in line with the previous animal and clinical studies [29]. Actually, both aerobic and resistance exercise have been shown to improve spatial learning and memory in both humans [30] and rodents [31]. Previous studies have also demonstrated that exercise-induced upregulation of autophagy can be found in a number of tissues, driving the beneficial effects of exercise on the cardiovascular system, hepatic metabolism [32], and aging [33]. Moreover, the common roles of CAT and HIIT in cardioprotection and neuroprotection have been widely reported, highlighting their roles in promoting the survival of cardiomyocytes and neuronal cells [34,35]. In addition to their common biological roles, the distinct roles of CAT- and HIIT-regulated EV miRNAs were also identified in our study. The CAT-regulated EV miRNAs were found to be involved in synaptic plasticity, memory, and substrate metabolism, which provides the molecular details of the protective roles of CAT against neurological and metabolic disorders [36,37]. Meanwhile, the HIIT-regulated EV miRNAs were more strongly associated with vascular endothelial growth, muscle function, and insulin signaling, which elucidates the underlying molecular mechanisms of certain reported beneficial roles of HIIT. Callahan et al. showed that HIIT contributes to increased muscle protein synthesis and muscle fiber size [38]. Furthermore, emerging evidence from human studies shows that high-intensity exercise results in improved insulin resistance and glucose homeostasis [39].

A large proportion of miRNAs are clustered in the genome, which can be commonly regulated and present similar expression patterns. It has been suggested that members of miRNA clusters can share the same target genes or regulate the genes that are involved in a specific pathway [40,41]. Interestingly, 11 members of the miR-379 cluster within plasma EVs were shown to be downregulated by HIIT, where these spatial neighboring miRNAs share the same promotor and collaborate in the regulation of specific cellular processes [40]. Actually, the miR-379 cluster is known for its impacts on neurodevelopment, tumor metastasis, hyper-glucocorticoidemia, and obesity [42]. In addition, Okamoto et al. reported that upregulated miR-379 is strongly associated with non-alcoholic fatty liver disease [43]. Our results show that the target genes of these DE miR-379 cluster members could interact with each other via multiple pathways, particularly the neuroactive ligand–receptor interaction pathway. Among the target genes of the DE miR-379 cluster members, POMC and GLP1R are particularly relevant to neural function and systemic energy metabolism [44,45]. GO pathway enrichment analysis also indicated that the HIIT-regulated miR-379 cluster may have an impact on biological processes such as glucose homeostasis and immunologic function.

In this study, we also performed a comprehensive analysis of the DE EV proteins. Notably, we found that most exercise-regulated circulating EV proteins are free of a predicted signal peptide sequence and are assumed not to be classically secreted proteins [8]. Exercise may probably serve as the driving force for protein encapsulation into EVs and the release of EVs containing non-secreted proteins. Interestingly, a portion of CAT- and HIIT-regulated EV proteins were commonly associated with vesicle secretion, transport, and localization, which is strikingly different from the common biological roles of CAT- and HIIT-regulated EV miRNAs. However, exercise-regulated EV proteins and EV miRNAs were also demonstrated to be involved in overlapping biological roles. For instance, CAT-regulated EV proteins were shown to be associated with organic substance and macromolecule metabolic processes involving carbohydrate and lipid metabolism, while HIIT-regulated EV proteins were suggested to be involved in cell death and survival, which is consistent with our previous results generated from DE EV miRNAs. Taken together, these data suggest that CAT-induced plasma EVs contribute to carbohydrate and lipid metabolism, while HIIT-induced plasma EVs are involved in cell death and survival, which is in line with clinical evidence showing that CAT promotes fat oxidation and insulin sensitivity [46], while HIIT prevents the apoptosis of skeletal muscle cells [47].

The choice of exercise type may depend on individual preferences, time availability, and physical fitness levels; however, certain recommendations can be achieved based on the current study. Given the common beneficial roles of CAT and HIIT in cardiomyocytes, it is suggested that individuals engaging in either type of exercise can achieve better cardiovascular function and a reduced risk of cardiovascular incidents. Individuals at risk for neurodegenerative diseases (e.g., Alzheimer’s disease) and those with metabolic diseases (e.g., non-alcoholic steatohepatitis [NASH] or diabetes) may benefit more from CAT due to the potential beneficial roles of CAT in neuronal function and metabolism. On the other hand, HIIT is recommended for individuals who seek to increase muscle mass and strength, as well as for those who aim to promote injury repair and enhance resilience to stress, considering the potential beneficial roles of HIIT in muscle function and cell viability.

Since a “humoral” factor with hypoglycemic properties was discovered to be released from skeletal muscles in response to exercise [48], the role of skeletal muscle as a secretory organ in mediating exercise-induced organ crosstalk has been heavily investigated. Growing evidence suggests that EVs containing bioactive molecules can be released from various tissues and play essential roles in tissue/organ crosstalk during exercise [49], which opens a new avenue for the studies of exercise-induced organ crosstalk. We therefore aimed to identify the tissue origin of DE plasma EV miRNAs and EV proteins. We found that numerous tissues contributed to exercise-induced alterations in the expression of EV contents. Surprisingly, a large portion of the DE EV miRNAs and EV proteins were found to be enriched in the nervous system. For instance, the top CAT-upregulated EV miR-124-5p is uniquely expressed in the nervous system. MiR-124 has been suggested to play critical roles in neuronal development and function, and dysregulation of miR-124 is associated with various neurological disorders, including Alzheimer’s disease, Parkinson’s disease, hypoxic–ischemic encephalopathy, Huntington’s disease, and ischemic stroke [50]. The top CAT-upregulated EV protein, the myelin sheath, functions as a crucial insulating membrane layer that envelopes myelinated axons in vertebrates, playing an important role in neural transmission [51]. Furthermore, the top HIIT-upregulated EV miRNA (miR-6511a-3p) and the top HIIT-downregulated EV miRNA (miR-137) were all found to be enriched in the nervous system. These data highlight the potential roles of nervous system-derived EVs in exercise-induced organ crosstalk. Interestingly, CAT-regulated EV miRNAs were shown to be enriched in multiple metabolic tissues, including the liver, pancreas, muscle, and adipocytes, which supports our previous observations on the roles of CAT in substrate metabolism. Additionally, both HIIT-upregulated EV miRNAs and EV proteins were found to be largely enriched in the immune system, which is in line with the reported immune regulatory roles of HIIT [13].

Admittedly, there are some limitations to our study. First, the number and diversity of participants in the current study were comparatively limited. This will not only reduce the statistical power of the findings but also affect the generalization of the results. Secondly, this study involves two types of exercise, and the varying workloads associated with different exercise modalities may potentially confound the results. Thirdly, the plasma EV samples used in this study were largely heterogeneous due to the limited isolation and analysis methods for EVs. Finally, the exact contributions of each tissue to the DE EV contents and their underlying mechanisms cannot be elucidated due to the lack of relevant methodologies.

To further validate our results, future research should include volunteers from diverse demographics, such as females, older adults, and individuals with various health conditions. And more exercise types with different workloads should also be considered. Meanwhile, circulating EVs provide direct and rapid responses to exercise, making them suitable for initial experimental exploration. The non-invasive samples such as urine, sweat, and tears also serve as excellent alternatives in future research, which will offer additional molecular insights into the role of exercise.

In summary, we provide the molecular details of the systemic effects of CAT and HIIT by analyzing the circulating EV contents. To our knowledge, no previous studies have compared different types of exercise using a multi-omics integration analysis of circulating EVs. We showed that CAT and HIIT could play common roles in neuronal signal transduction, autophagy, as well as cell fate regulation. CAT also plays distinct roles in cognitive function and substrate metabolism, while HIIT is strongly associated with muscle performance, insulin signaling, and positive regulation of overall cell function. It is postulated that EX-EVs likely originate from various tissues, including metabolic tissues, immune systems, and the largely neglected nervous system. This study provides the basis for a better understanding of exercise-mediated organ crosstalk and its potential health-promoting roles.

## 4. Materials and Methods

### 4.1. Study Design and Participants

In total, five healthy male volunteers were enrolled in the study. The inclusion criteria were as follows: (1) 18–65 years of age; (2) body mass index (BMI) values between 18 and 28 kg/m^2^; (3) more than 3 h of physical activity per week; (4) acknowledgment of informed consent. The exclusion criteria were as follows: (1) smokers; (2) body weight change >5 kg in 6 months; (3) unsuitable for physical training (heart disease, respiratory disorders, or any conditions that could be aggravated by exercise); (4) currently taking medication or having a history of medication such as steroids, beta-blockers, or anticoagulants.

Before the formal experiment, the volunteers underwent a thorough physical examination (height, weight, body fat percentage, heart rate, and blood analysis) and proper adaptive training for the experimental protocol to ensure that they were able to complete the experiment. The participants refrained from exercise 24 h prior to the test to ensure the integrity and accuracy of the results. All volunteers had the same breakfast, and all tests started at 9:00 a.m. The volunteers successively completed both HIIT and CAT under the supervision of a professional coach, and the interval between each type of exercise was 7 days. Two exercise types have the same total time. Each training session was initiated with a brief 2 min of dynamic stretching to warm-up, followed by 20 min of cycling consisting of periods of 2 min at 80–95% maximal heart rate (HRmax) separated by 2 min of active recovery for the HIIT group or 20 min of cycling at 60–80% of HRmax for the CAT group. A real-time heart rate monitoring system was continuously used during each training session. An HRmax was estimated using the age-predicted equation of 220–age [52]. The participants were instructed to continue their regular physical activities and eating habits throughout the intervention period (Figure 1A). Blood samples were collected at rest or immediately after each training session for further analysis. Informed consents were obtained from all participants, and the experimental procedures were approved by the Ethics Committee of the West China Hospital of Sichuan University (approval No. 2022629).

### 4.2. Plasma EV Isolation

Blood was collected in heparin-coated blood collection tubes (avoiding excessive agitation) and immediately centrifuged at 1600× *g* for 10 min at RT. Afterward, the supernatant was carefully collected from the top down with a pipette, ensuring that a specified amount of the supernatant was left on top of the pellet [16]. Two milliliters of collected supernatant was centrifuged at 10,000× *g* for 30 min at 4 °C in a fixed-angle rotor (model 220.78, Hermle, Wehingen, Germany), followed by two washes with iced PBS to eliminate soluble proteins. The obtained pellet was resuspended in 1.5 mL of iced PBS and filtered through 0.2 μm syringe filters (Millex-GP; Merck Millipore, Darmstadt, Germany). Then, the final volume of the filtrate was top-up with iced PBS to 1.5 mL prior to centrifugation at 47,000 rpm [RCF (average) 98,963, RCF (maximum) 130,000, k-factor 90.4] for 2 h at 4 °C in a Beckman TLA-55 rotor (Beckman Coulter, Krefeld, Germany). Finally, the pellets were resuspended in iced PBS, aliquoted in Eppendorf Polyallomer tubes, and stored in a −80 °C freezer, with care taken to avoid repeated freeze–thaw cycles during analysis.

### 4.3. Characterization of EVs

Specific EV markers and the proper controls were analyzed via Western blotting, as previously described [53]. TSG101 (Cell Signaling Technology, #72312, 1:1000), CD9 (Abcam, ab307085, 1:1000), and CD81 (Abcam, ab79559, 1:1000) were chosen as EV-positive markers. Calnexin (Cell Signaling Technology, #2433, 1:1000) was chosen as an EV-negative marker, and apolipoprotein AI (Abcam, ab7613, 1:1000) was chosen as a positive marker of plasma.

The size distribution and particle concentration of the EVs were analyzed using nanoparticle tracking analysis (NTA) instrument ZetaView PMX120 (Particle Metrix, Inning am Ammersee, Germany). For each measurement, five consecutive NTA videos were captured across all 11 positions at room temperature. The analysis parameters were set as follows: sensitivity = 75, shutter speed = 75, minimum brightness = 20, and minimum detectable particle size = 5 nm.

The morphology of the EVs was examined by transmission electron microscopy (TEM, model HT7800, Hitachi, Ltd., Tokyo, Japan). Specifically, 10 μL of a diluted EV solution was applied to a carbon-supported copper grid and subsequently subjected to negative staining with a 2% phosphotungstic acid solution. Following air drying at ambient temperature, the grids were analyzed using TEM at a voltage of 80 kV [53].

### 4.4. EV RNA Extraction and miRNA Sequencing

Total RNA, including small RNAs, was extracted from EVs using the miRNeasy kit (Qiagen, Hilden, Germany) according to the manufacturer’s instructions (Table S2). The miRNA library was constructed using the NEBNext Multiplex Small RNA Library Prep Set for Illumina (catalog #E730, New England Biolabs), according to the manufacturer’s instructions. A unique molecular identifier provided by Seqhealth Technology Co., LTD was utilized to label the pre-amplified small RNA molecules. The RNA library was purified through 6% polyacrylamide gel electrophoresis. Library quantification was performed using a QubitTM3 fluorometer (Invitrogen, catalog# Q33216) along with the Qubit dsDNA HS Assay Kit (Invitrogen, catalog # Q32854). The quality of the library was assessed using the Qsep100TM bio-fragment analyzer (Bioptic Inc., New Taipei City, Taiwan, Changzhou, China). The RNA library was sequenced on a Novaseq 6000 sequencer (Illumina) with a PE150 model. The raw sequencing data were filtered to remove low-quality reads using the FASTX-Toolkit (version 0.0.13.2), and the adaptor sequences were trimmed using cutadapt (version 1.15). Processed reads were then treated to minimize duplication bias.

For the miRNA sequencing data analysis, the clean read sequences were aligned against the Silva, GtRNAdb, Rfam, and Repbase databases using Bowtie software. This process served to filter out ribosomal RNA (rRNA), transfer RNA (tRNA), small nuclear RNA (snRNA), small nucleolar RNA (snoRNA), and other non-coding RNAs (ncRNAs), as well as repeats. The sequences that remained after filtering were then compared to known miRNAs from miRbase and the Human Genome (GRCh38) to identify both known and predicted novel miRNAs. Read counts for each miRNA were extracted from the mapping results, and transcripts per million (TPM) were calculated. Comparison between the two sets of replicate samples was conducted using the limma R package [14].

### 4.5. Protein Extraction and Proteomic Profiling of EVs

EV samples were lysed using RIPA buffer (catalog# 89901, Thermo Scientific, Waltham, MA, USA) supplemented with Halt™ protease inhibitor mixture (catalog# 87785, Thermo Scientific, Waltham, MA, USA), followed by extensive sonication in an ice bath. The lysate was then centrifuged at 20,000× *g* for 20 min at 4 °C, and the supernatant was carefully collected and transferred to a sterile EP tube. Next, the samples were reduced with 10 mM DTT for 1 h at 56 °C and subsequently alkylated with iodoacetamide for 1 h at room temperature in a dark environment. This was followed by mixing the samples with 4 volumes of acetone and incubating them at −20 °C for 2 h. After centrifugation, the resulting pellet was washed with cold acetone and solubilized in 0.1 M of TEAB containing 6 M of urea. The protein concentration of the samples was determined using the PierceTM BCA Protein Assay Kit (catalog# 23,225, Thermo Scientific, Waltham, MA, USA).

For LC-MS/MS analysis, the lyophilized samples were dissolved in a 0.1% formic acid solution (referred to as solvent A). These dissolved samples were then injected into a C18 Nano-Trap column. Peptide separation occurred within an analytical column using a mobile phase consisting of 0.1% formic acid in 80% acetonitrile (termed solvent B). The elution process involved gradually increasing the concentration of solvent B from 6% to 100% over a 60 min period while maintaining a constant flow rate of 600 nL/min.

The separated peptides were subsequently injected into a Nanospray Flex ESI source with a spray voltage of 2.3 kV and analyzed using an Orbitrap Exploris 480 mass spectrometer (Thermo Fisher, Waltham, MA, USA). The raw data from the mass spectrometry assays were searched against the UniProt database. During this search, carbamate was set as a fixed modification, while methionine oxidation (M) and N-terminal acetylation were designated as variable modifications. Label-free protein quantification was performed using Proteome Discoverer software version 2.2.

### 4.6. Bioinformatic Analysis

The screening criteria for the DE miRNAs and proteins were |log2(FC)| ≥ 0.5 and *p* value ≤ 0.05. The miRNA targets were predicted using RNAhybrid [53] and miRanda [54]. A Venn diagram was generated to visualize the overlapping target genes. The protein–protein interaction (PPI) network was constructed based on the STRING database [55] involving predicted and experimentally verified protein interactions. Subsequent analysis was performed using Cytoscape software, which also facilitated the identification of hub proteins within the network. The TAM 2.0 database was used to determine the miRNA clusters [56]. Signal peptides of proteins were identified using SignalP 5.0 [57], and protein subcellular localization was predicted based on Hum-mPLoc 3.0 [58]. Tissue enrichment analysis of miRNAs and proteins was performed using the Tissue Atlas [59] and Human Protein Atlas, respectively. The significance A/B method was used to calculate the significance of the difference between samples [60]. All bioinformatics calculations were further processed using R software (version 4.3.0). The repeated samples between groups were analyzed using the limma R package. The heatmaps were created using the Complex Heatmap package (v. 2.14.0), the correlation was calculated by the corrplot package (v. 0.95), the Volcano plot was created using the ggplot2 package (v. 3.5.0), and the Sankey diagram was created using the ggalluvial package (v. 0.12.5). All analysis tools used in this study are summarized in Table S4.

### 4.7. GO and KEGG Pathway Enrichment Analysis

Functional annotation and pathway enrichment analyses were conducted utilizing the Cluster Profiler package [61] with Gene Ontology (GO) terms [62] and Kyoto Encyclopedia of Genes and Genomes (KEGG) pathways [63] as reference datasets.

### 4.8. Statistical Analysis

Statistical analyses were conducted with GraphPad Prism 8.0 (GraphPad Software, San Diego, CA, USA). Each experiment of EV characterization was replicated a minimum of three times. The results are presented as the mean ± standard error of the mean (SEM) and compared using Student’s *t*-test. All statistical analyses were performed with GraphPad Prism (8.0), and *p* < 0.05 was considered statistically significant.

## Data Availability

The data that support the findings of this study are available from the corresponding author upon reasonable request.

## Supplementary Materials

The following supporting information can be downloaded at: https://www.mdpi.com/article/10.3390/ijms26031383/s1.
