# Supplementary material for: Differential Regulation of miRNA and Protein Profiles in Human Plasma-Derived Extracellular Vesicles via Continuous Aerobic and High-Intensity Interval Training
Source: Int J Mol Sci. 2025 Feb 6;26(3):1383. doi: 10.3390/ijms26031383 (PMC11818269; doi:10.3390/ijms26031383)
Supplement: Supplementary file 1 [file ijms-26-01383-s001.zip › ijms-3404479-supplementary.pdf]

# Supplementary Materials for

# Differential regulation of miRNA and protein profiles in human plasma-derived extracellular vesicles by continuous aerobic and high intensity interval training

Zhenghao Wang[1,2,#], Yiran Ou[1,#], Xinyue Zhu[1], Ye Zhou[1], Xiaowei Zheng[1,3], Meixia Zhang[4], Sheyu Li[1], Shao-Nian Yang[2], Lisa Juntti-Berggren[2], Per-Olof Berggren[1,2,¶], Xiaofeng Zheng[1,¶,*]

[1]Department of Endocrinology and Metabolism, Research Center for Islet Transplantation, West China Hospital, Sichuan University, Chengdu 610041, China

[2]The Rolf Luft Research Center for Diabetes and Endocrinology, Karolinska Institutet, SE-17176 Stockholm, Sweden

[3]Department of Molecular Medicine and Surgery, Karolinska Institutet, SE-17177 Stockholm, Sweden

[4]Department of Ophthalmology and Research Laboratory of Macular Disease, West China Hospital, Sichuan University, Chengdu 610041, China

[#]These authors share co-first authorship

[¶]These authors share co-senior authorship

*Correspondence: Xiaofeng Zheng (xiaofeng.zheng@wchscu.cn)

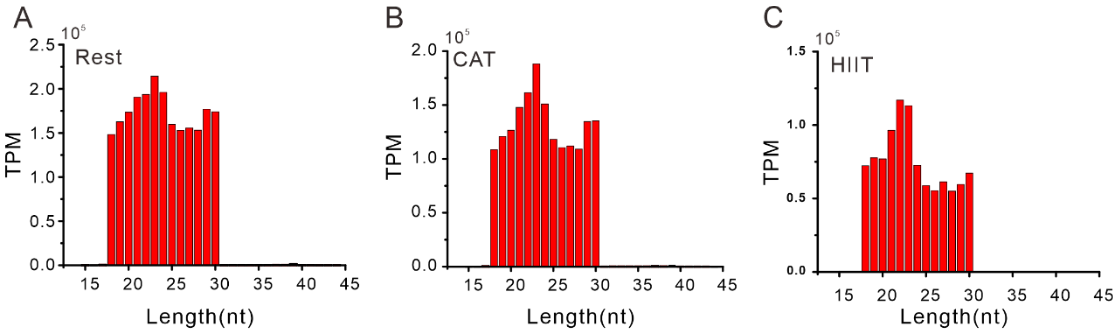


**Figure S1. The length distributions of the EV-miRNAs in the three study groups.**

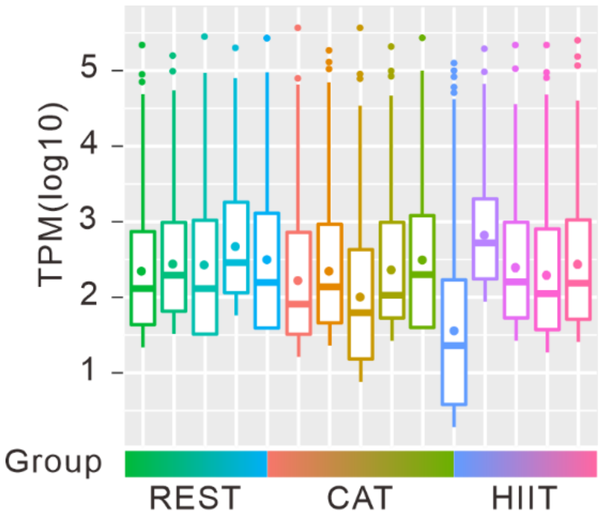


**Figure S2. The transcripts per Kilobase Per Million mapped read (TPM) of EV-miRNAs in all tested samples.**

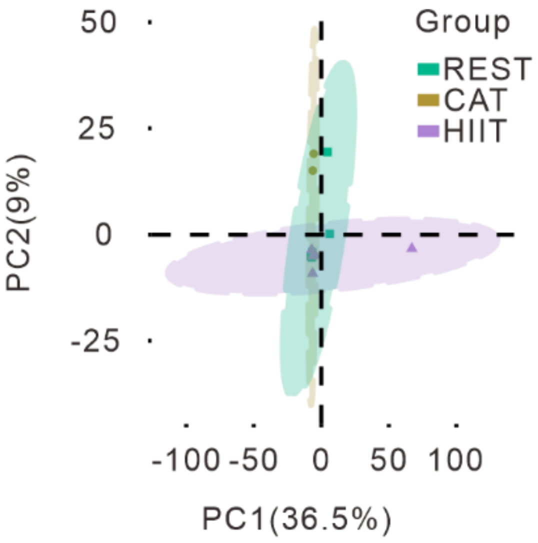


**Figure S3. PCA of miRNA expression profiles in the three study groups.**

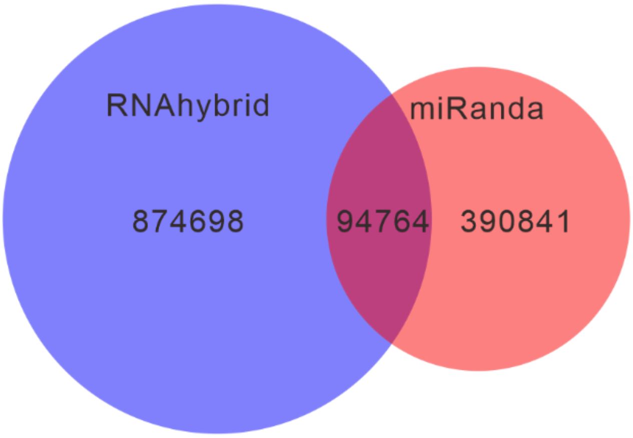


**Figure S4. Venn diagram of predicted target genes of the DE EV-miRNAs based on RNAhybrid database and miRanda database.**

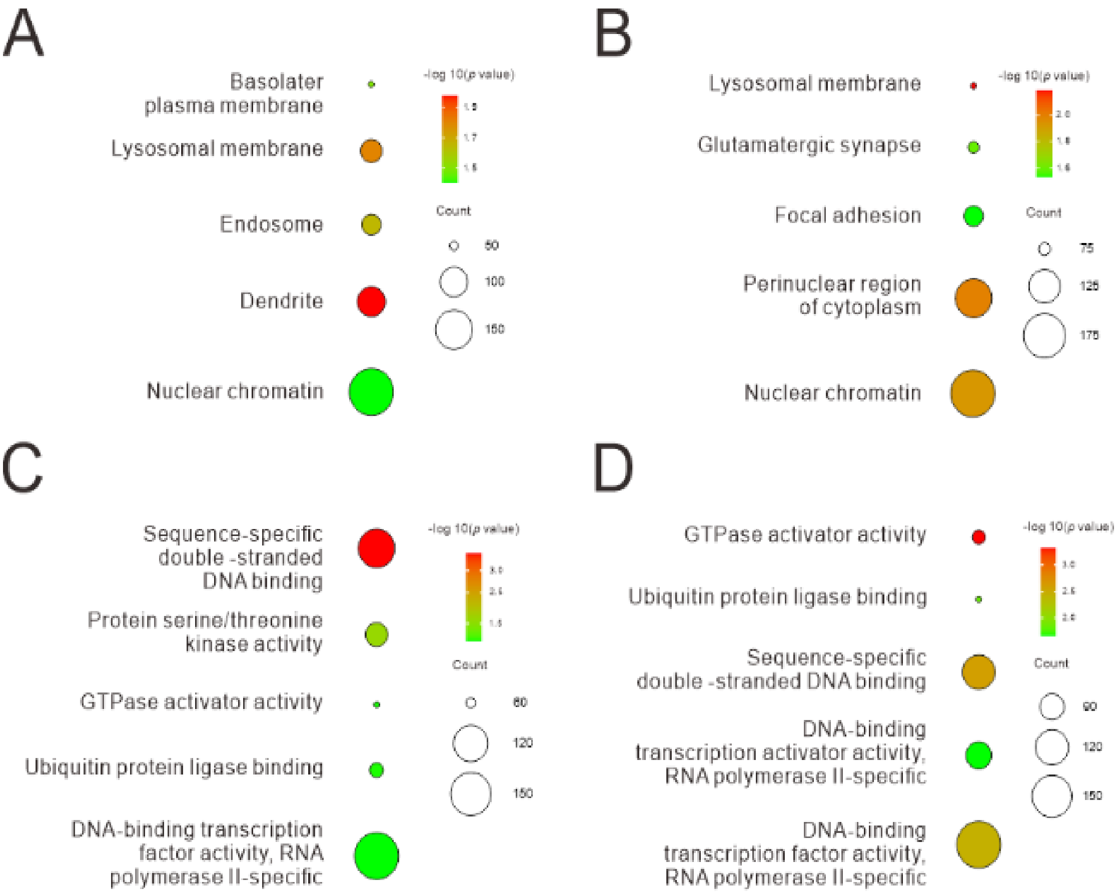


**Figure S5. GO Enrichment analysis of the DE EV-miRNAs.** (A) Enrichment analysis for GO-CC (the CAT group vs the Rest group). (B) Enrichment analysis for GO-CC (the HIIT group vs the Rest group). (C) Enrichment analysis for GO-MF (the CAT group vs the Rest group). (D) Enrichment analysis for GO-MF (the HIIT group vs the Rest group).

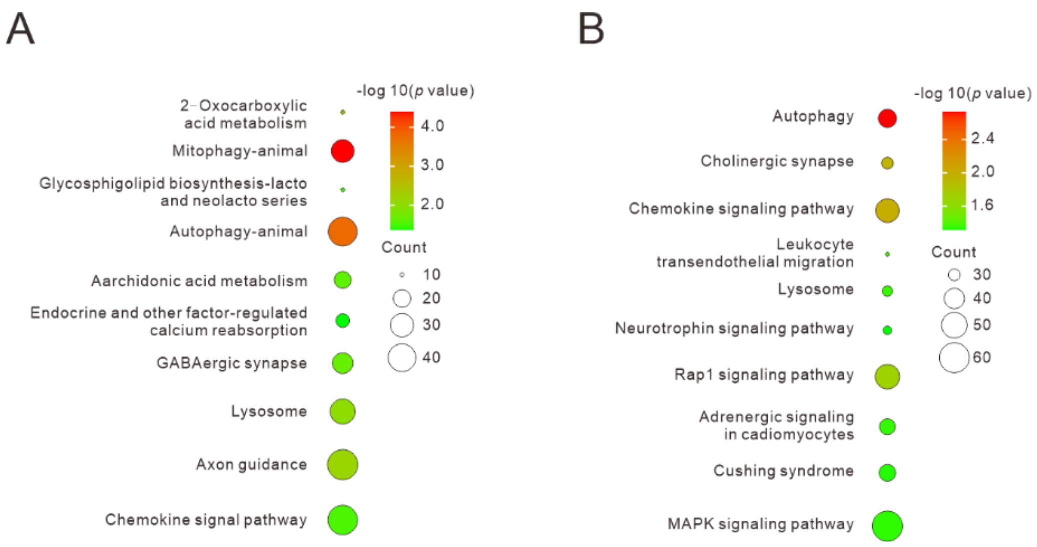


**Figure S6. KEGG pathway enrichment analysis of the DE EV-miRNAs.** (A) the CAT group vs the Rest group. (B) the HIIT group vs the Rest group.

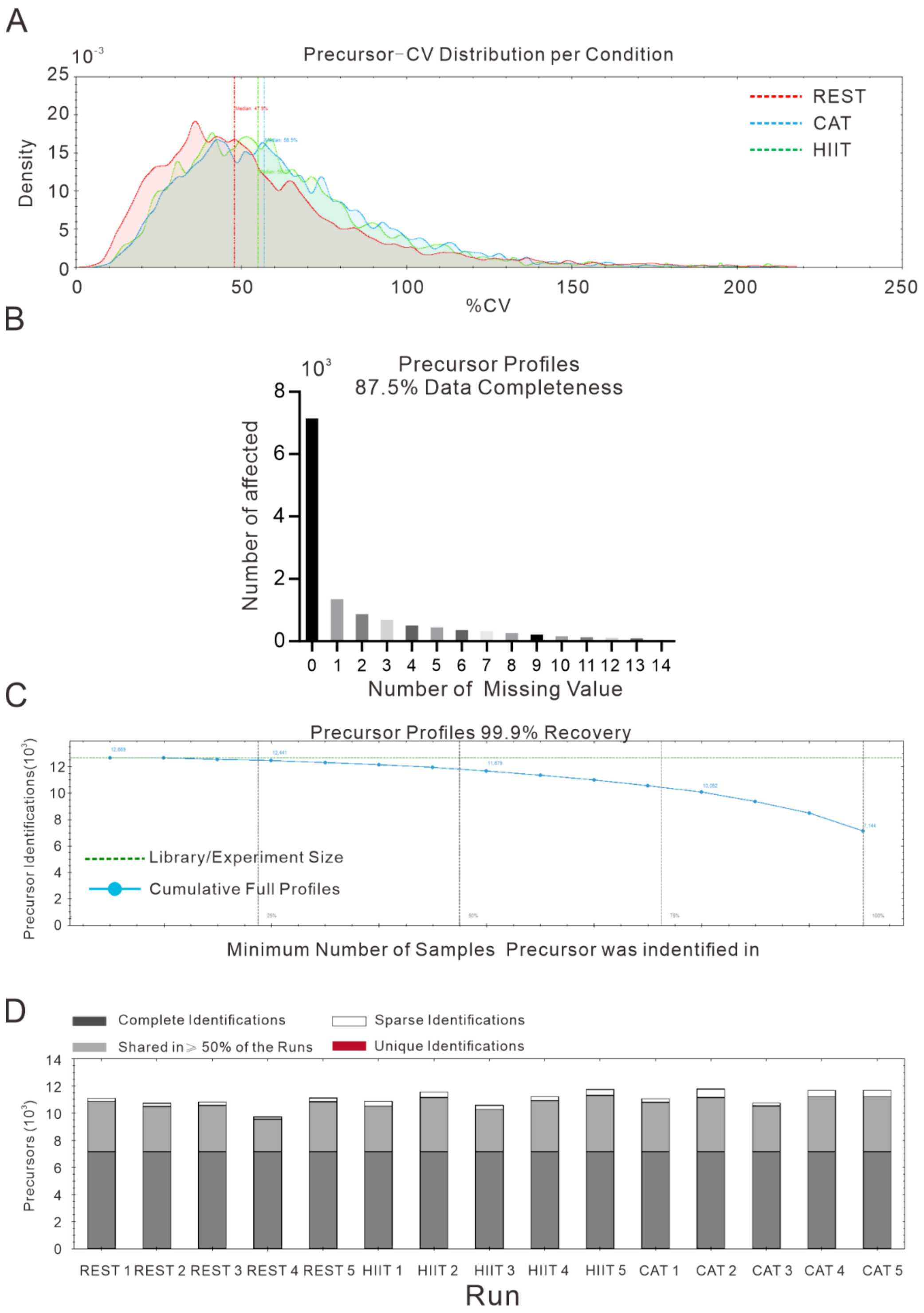


**Figure S7. The quality control (QC) assessment of proteomics data.** (A) coefficient of variation, (B) data completeness, (C) reproducibility and(D) run identification of proteomics data.

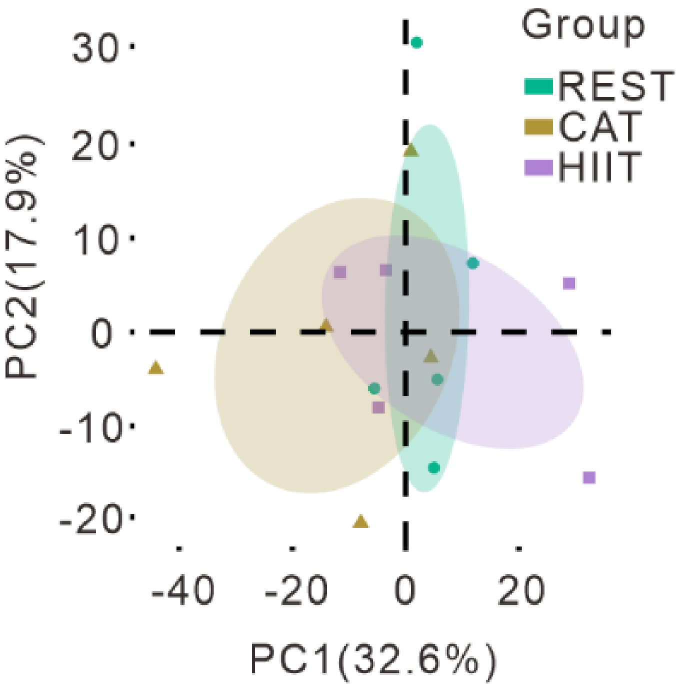


**Figure S8. PCA of proteomic profiles in the three study groups.**

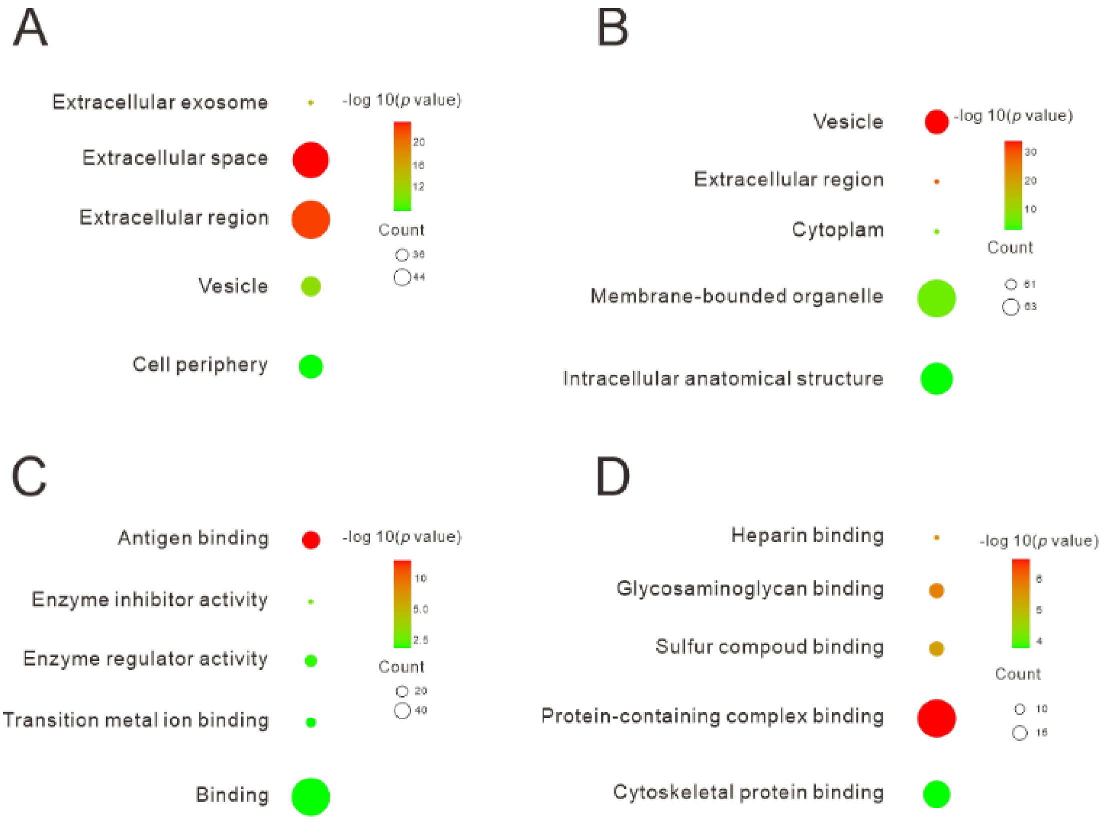


**Figure S9. GO Enrichment analysis of the DE EV-proteins.** (A) Enrichment analysis for GO-CC (the CAT group vs the Rest group). (B) Enrichment analysis for GO-CC (the HIIT group vs the Rest group). (C) Enrichment analysis for GO-MF (the CAT group vs the Rest group). (D) Enrichment analysis for GO-MF (the HIIT group vs the Rest group).

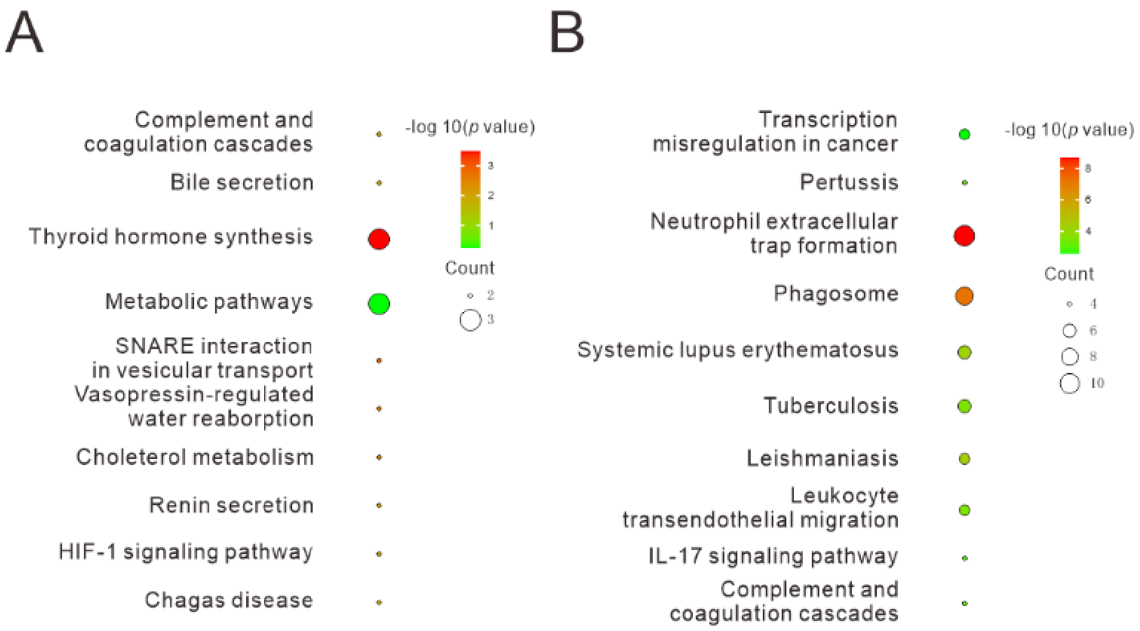


**Figure S10. KEGG pathway enrichment analysis of the DE EV-proteins.** (A) the CAT group vs the Rest group. (B) the HIIT group vs the Rest group.

**Table S1. Clinical characteristics of exercise volunteers.**

BMI: body mass index, WHR: waist hip rate, ALT: alanine aminotransferase, AST: aspartate aminotransferase, Crea: creatinine, FBG: fasting blood glucose, TG: triglyceride, HDL-C: high density lipoprotein cholesterol, LDL-C: low density lipoprotein cholesterol, BFP: body fat percentage

| n | 5 |
|---|---|
| Sex | Male |
| Age (year) | 29.4±1.34 |
| BMI (kg/m$^2$) | 24.92±2.16 |
| WHR (%) | 0.884±0.04 |
| ALT (IU/L) | 22.8±11.12 |
| AST (IU/L) | 21±7.61 |
| Crea (umol/L) | 90±6.48 |
| FBG (mmol/L) | 4.732±0.43 |
| TG (mmol/L) | 1.358±0.51 |
| HDL-C (mmol/L) | 1.238±0.13 |
| LDL-C (mmol/L) | 2.74±0.396 |
| BFP (%) | 24.04±5.18 |

**Table S2. miRNA quantities of serum EV samples.**

| Samples | RNA concentration (ng/μL) | RNA amount (ng) |
|---|---|---|
| REST 1 | 0.39 | 10.92 |
| REST 2 | 0.31 | 8.68 |
| REST 3 | 0.23 | 6.44 |
| REST 4 | 0.41 | 11.48 |
| REST 5 | 0.49 | 13.72 |
| CAT 1 | 0.40 | 11.2 |
| CAT 2 | 0.15 | 4.2 |
| CAT 3 | 0.25 | 7 |
| CAT 4 | 0.54 | 15.12 |
| CAT 5 | 0.44 | 12.32 |
| HIIT 1 | 0.32 | 8.96 |
| HIIT 2 | 0.26 | 7.28 |
| HIIT 3 | 0.21 | 5.88 |
| HIIT 4 | 0.50 | 14 |
| HIIT 5 | 0.32 | 8.96 |

**Table S3. The unique and overlapping GO terms of CAT group and HITT group.**

| CAT | Common | HIIT |
|---|---|---|
| Regulation of triglyceride metabolic process | | Glycerophospholipid metabolic process |
| Cholesterol metabolic process | | Positive regulation of insulin secretion |
| Cardiac conduction | | Positive regulation of autophagy |
| Renal water homeostasis | | Actin cytoskeleton organization |
| Neural tube development | | Homophilic cell adhesion via plasma membrane adhesion molecules |
| Cellular response to mechanical stimulus | | Intracellular protein transport |
| Cell volume homeostasis | | Actin filament organization |
| Cation transport | | Adenylate cyclase-modulating G protein-coupled receptor signaling pathway |
| Protein phosphorylation | | Regulation of cell shape |
| Transmembrane receptor protein tyrosine kinase signaling pathway | Autophagy | Protein maturation |
| Signal transduction | Macroautophagy | Negative regulation of transcription by RNA polymerase II |
| Cellular response to peptide | Negative regulation of cell population proliferation | Regulation of nucleic acid-templated transcription |
| Cellular ion homeostasis | Positive regulation of cell differentiation | Response to cAMP |
| Cell-cell junction assembly | | Positive regulation of MAPK cascade |
| Positive regulation of calcium ion transport into cytosol | | Positive regulation of transcription by RNA polymerase II |
| Cation transmembrane transport | | Regulation of macroautophagy |
| Hemostasis | | Negative regulation of ERK1 and ERK2 cascade |
| Protein autophosphorylation | | Regulation of actin cytoskeleton organization |
| Positive regulation of cell adhesion | | Wnt signaling pathway |
| | | Positive regulation of protein kinase activity |
| | | Regulation of ion transmembrane transport |
| | | Response to hypoxia |
| | | Exocytosis |
| | | Regulation of sodium ion transport |
| | | Intracellular signal transduction |

**Table S4. List for the online analysis tools.**

| Name of the analysis tools | Website |
|---|---|
| Ahybrid | http://bibiserv.techfak.uni-bielefeld.de/rnahybrid |
| Randa | http://www.microrna.org/ |
| STRING | http://string-db.org |
| TAM 2.0 | http://www.lirmed.com/tam2/ |
| SignalP 5.0 | https://services.healthtech.dtu.dk/services/SignalP-5.0/ |
| Hum-mPLoc3 | http://www.csbio.sjtu.edu.cn/bioinf/Hum-mPLoc3/ |
| Tissue Atlas | https://ccb-web.cs.uni-saarland.de/tissueatlas2 |
| Human protein atlas | https://www.proteinatlas.org |
| GO analysis | https://www.geneontology.org/ |
| KEGG analysis | https://www.genome.jp/kegg/ |
